# Visual recognition memory does not benefit from preserved temporal order from encoding to test

**DOI:** 10.3758/s13414-026-03307-7

**Published:** 2026-07-16

**Authors:** Chong Zhao, Keisuke Fukuda, Geoffrey F. Woodman

**Affiliations:** 1 Department of Psychology, University of Chicago, Chicago, IL 60637 USA; 2 Institute for Mind and Biology, University of Chicago, Chicago, IL 60637 USA; 3 Department of Psychology, University of Toronto Mississauga, Mississauga, Ontario L5L 1C6 Canada; 4 Department of Psychology, University of Toronto, Toronto, Ontario M5S 3G3 Canada; 5 Department of Psychology, Vanderbilt Vision Research Center, Vanderbilt University, PMB 407817, 2301 Vanderbilt Place, Nashville, TN 37240-7817 USA

**Keywords:** Temporal context, Temporal clustering, Visual memory, Long-term memory, Object memory, Serial order

## Abstract

Research suggests that items from similar spatiotemporal contexts are more likely to be retrieved together. Here, we sought to test whether preserving temporal order from encoding to test might lead to a retrieval benefit. Then, we tested the participants using test items that were either presented in the same order as during encoding, or the order of items was different due to it being randomized. Across two experiments, we found that maintaining the same temporal order did not improve overall memory performance in the visual old/new recognition task we used. Next, we show that conditional-response probability plots demonstrate temporal grouping from the study order, but the strength of that grouping was unmodulated by whether the same order or a random order was used during recognition testing. Lastly, we used a two-alternative forced-choice task and again found that presenting old items in their original order, without intervening new items, did not enhance recognition memory performance. Thus, we find that for visually presented common objects tested with recognition, we do not see that the same order boosts participants’ ability to retrieve a sequence of visual objects from memory.

## Introduction

Humans possess an extensive visual memory capacity. Research has demonstrated that individuals can recognize thousands of scenes, objects, or spatial locations of objects after just a few seconds of exposure (Lionel, 1973; Wolfe et al., 2023). However, the accuracy of a participant’s memory appears to vary from moment to moment based on several factors. Some of these factors are endogenous to the individual and beyond the experimenters’ control, although we can measure their fluctuations using brain activity (e.g., Fukuda & Woodman, 2015). One exogenous factor that people believe is critical for memory is the temporal structure of events (Healey et al., 2019; Polyn et al., 2009; Squire et al., 2004). In particular, memory for the temporal order of stimuli is typically enhanced when the items are encoded within the same context (DuBrow & Davachi, 2013), such as a sequence of visual images in recognition memory tasks. Here, we sought to test a simple prediction of these accounts that visual events are easier to pull out of memory if the events happening in the same order at test as was experienced at study.

The existing literature suggests that it should be possible to observe the influence of study–test temporal congruity of memory for visual images in the laboratory. Specifically, one study using 64 images as study materials showed that when the test images were adjacent to each other during encoding, their test accuracies were much higher than if they were remote to each other during encoding (Schwartz et al., 2005). Theoretically, when participants encode neighboring items during the study phase, they form either item–item associations (Lewandowsky & Murdock, 1989; Murdock, 1982  3) or item–context associations (Polyn et al., 2009). At test, presenting one item can cue retrieval of its neighbors through these associations, which should, in principle, enhance memory for items that were originally encoded in the same *temporal order*. These findings support the *temporal order benefit hypothesis*, which suggests that matching temporal contexts should facilitate memory retrieval (Howard & Kahana, 2002; Polyn et al., 2009). During the test phase, when participants successfully retrieved the learnt item with high confidence, they were also more likely to retrieve its temporal neighbor they had experienced in the stream of stimuli. Building on prior work showing that shared context enhances retrieval, we asked whether *temporal order* alone is sufficient to confer a memory benefit via item–item associations.

To test the temporal order benefit hypothesis in the present study, we asked the participants to encode 100 highly distinctive images of real-world objects. To experimentally test the effect of context on human recognition memory, half of the participants received a test list, where all the old items followed the same presentation order as during encoding. The other half of the participants received a randomized order of test items that was different from its encoding order. The *temporal order benefit hypothesis* predicts that the overall accuracy would be higher for the test list following the same order as encoding than the list following a different order. These benefits should extend beyond this global level to the local stimulus sequences. For each item position, the test list that followed the same order as encoding would have stronger contextual support than the random order, resulting in higher conditional-response probabilities surrounding correctly remembered items compared with the baseline with the randomized order. To eliminate the effects of stimulus-specific idiosyncrasies within each stimulus set (i.e., memorability; Bainbridge, 2017; Zhao et al., 2024a, 2024b), we administered two experiments where the studied items used in Experiment 1 served as the new items during test used in Experiment 2, and the new items used in Experiment 1 served as the studied items used in Experiment 2.

## Methods

### Experiment 1

We first estimated the power necessary to detect differences between groups using long-term memory recognition tests. Using an estimated correlation rating of 0.5 derived from previous studies of visual memory (Zhao et al., 2024a, 2024b), and significance level of 0.05, we estimated that we needed 29 participants to achieve power of 0.8 in our experimental design (Faul et al., 2007). We collected data from 50 participants each condition, a total of 200 participants living in the USA, 18–35 years of age, using the Prolific online system. Participants were compensated at a rate of USD$12 for an hour of their time.

**Fig. 1:**
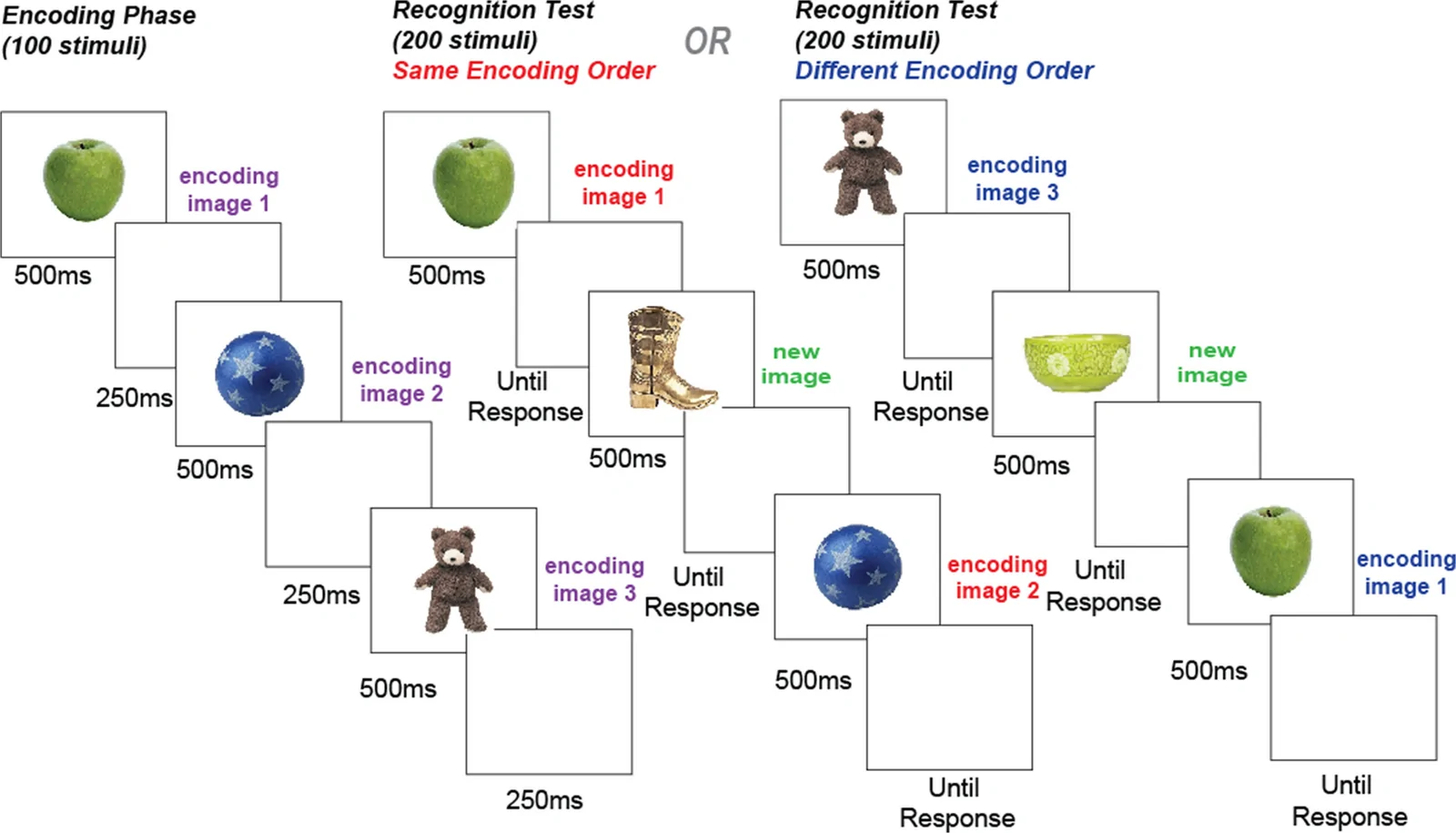
Fig. 1 The visual recognition memory paradigm we used in Experiments 1 and 2. Two-hundred adult participants were asked to first encode 100 pictures (one exemplar each from 100 semantic categories, Encoding Phase, left panel). They were later tested using recognition for the 100 studied pictures and 100 new pictures of exemplars from new semantic categories (one exemplar each from 100 new semantic categories). The critical between-subject manipulation was whether the studied images in test phase followed the same temporal order as during encoding phase. Half of the participants were assigned to the “same encoding order” group (middle panel), meaning that studied images in the test list followed the same order as in the encoding phase. The other half of participants were assigned a different fixed randomized order (right panel), such that the studied images followed a randomized order different from the encoding phase

After informed consent was obtained for procedures approved by the Vanderbilt University Institutional Review Board, every participant completed a picture-study phase followed by a recognition-memory test phase (see Fig. 1), using our standard visual long-term memory testing procedures (Fukuda & Woodman, 2015; Zhao et al., 2022; Zhao & Woodman, 2020).

During the study phase, we presented 100 photographs of real-world objects, with one exemplar from each of 100 distinct semantic categories. Mean picture size was approximately 4.6° × 4.6° of visual angle assuming the participant was seated 80 cm from the screen. Each image was centered on the screen during both study and test phases.

Each trial of the study phase started with a 1,000-ms fixation cross. Next, a picture was shown at the center of the screen for 250 ms, followed by another blank interval and then another picture, until all 100 pictures were shown.

During the test phase, we showed each participant 200 pictures, one at a time, with half of the pictures having been shown in the study phase and the other half being novel to the participant. Each trial of the test phase started with a 1,000-ms fixation cross, followed by the presentation of the test picture at the center of the screen until the participant pressed a button to record their old versus new response, as well as confidence level. The 100 novel lures had one exemplar each from 100 distinct categories; these categories were novel and were not used in the study phase.

We divided our 100 participants into two groups, consisting of 50 participants each. Both groups used the same 100 images (100 categories × 1 exemplar each) as their encoding set and shared the same 100 new images as the lures in their test phase. To manipulate the order of encoding, we ensured the target images in test phase followed the same order as in the encoding phase. Whereas for the other group, we ensured the target images in the test phase were in a fixed, randomized order, different from the order during encoding.

Participants used the numbers on a keyboard to indicate their confidence and whether they thought the test stimulus was old or new. The number keys 1 and 2 indicated that the item was old, with high and low confidence levels, respectively. The number keys 9 and 8 indicated that the item was new, with high and low confidence level, respectively. The stimuli were drawn from a published set of real-world objects (Brady et al., 2008), and programmed using the *jsPsych* package (de Leeuw, 2015).

### Experiment 2

As in Experiment 1, we collected data from 100 participants living in the USA, 18–35 years of age, using the Prolific online system. Each participant completed a picture-study phase followed by a recognition-memory test phase, using our standard visual long-term memory testing procedures, as in Experiment 1. Identical to Experiment 1, we divided our 100 participants into two groups consisting of 50 participants each. Both groups saw the same 100 images as in their encoding set (100 categories with one exemplar each) and also saw the same 100 new images as lures in their test phase. In Experiment 2 we used the encoding set of stimuli from Experiment 1 as the new lure images, whereas the new lure images from Experiment 1 were the old items in the encoding set in Experiment 2. This controls for potential effects of using specific images; the only difference between conditions is that in one group, the target images in the test phase followed the same order as in the encoding phase, whereas for the other group, the target images in the test phase were in a fixed, randomized order, different from the order used during encoding.

### Data analyses

In our analysis, we first calculated the accuracy, hit rate (HR), and false-alarm rate (FAR) for each participant. To further quantify the discrimination capability and response bias, we calculated *d* prime (*d*’) and beta (*β*) criterion values. The *d*-prime value was determined by converting the hit rate (proportion of correctly identified old items) and false-alarm rate (proportion of new items incorrectly identified as old) to *z* scores and computing their difference (*d*′ = Z(HR) − Z(FAR)). The beta criterion was calculated using the formula *β* = *ϕ*(Z(HR)/*ϕ*(Z(FAR), where ϕ denotes the probability density function of the standard normal distribution.

To provide an additional way of analyzing our data to determine whether following the same temporal order benefitted memory, we conducted a Receiver Operating Characteristic (ROC) analysis to evaluate the discriminative ability of participant responses in distinguishing between old and new items in the studied list (Van Zandt, 2000). The ROC curve was generated by plotting the true positive rate (sensitivity) against the false positive rate (1-specificity) at various threshold settings. Participant responses were categorized into four confidence levels: confidently old (1), somewhat old (2), somewhat new (8), and confidently new (9), and were mapped to continuous confidence scores for the analysis.

Finally, we investigated whether the retrieval of temporal context enhanced recognition for neighboring items during test. If so, when participants successfully retrieve an item with high confidence, they also should retrieve the associated temporal context from encoding. Thus, items adjacent to this high-confidence hit, which shared a similar temporal context during encoding, should be more likely to be successfully retrieved as well. Consequently, according to this *temporal order benefit hypothesis,* a list presented in the same order during both encoding and testing should benefit more from the consistent overlap between the encoding and retrieval context than a list presented in a different order, as each old item was at a +1 distance from the preceding item (Kahana & Howard, 2005). To test this prediction, we first compared the overall recognition memory performance between the same order group and the different order group. Subsequently, we also plotted the accuracies of neighboring items around each high-confidence hit, with the prediction being that all items, or at least those in the forward direction, would exhibit higher accuracy if the study–test temporal order was preserved compared with when it differed.

### Experiment 3

**Fig. 2:**
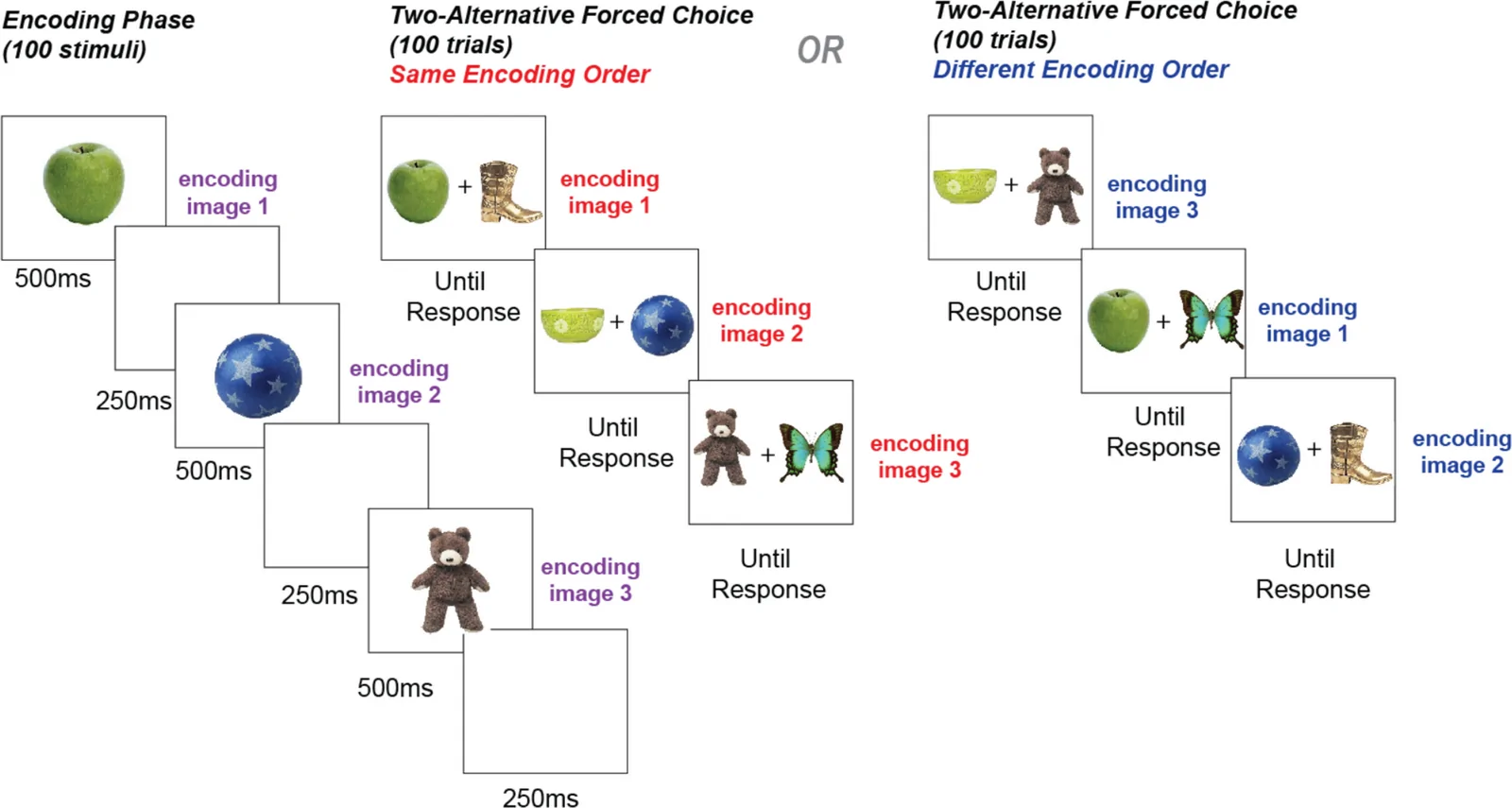
Two-alternative forced-choice (2AFC) visual recognition memory paradigm we used in Experiment 3. One hundred adults encoded 100 pictures, each from a different semantic category (left panel). They were later tested with a 2AFC task containing the 100 studied images and 100 new images from 100 new categories. Each test trial presented one old and one new picture, with the old image appearing equally often on the left and right. Participants selected which image was previously studied, allowing us to remove any influence of intervening new items present in standard old/new recognition tasks. The key between-subjects manipulation was the test order. Half of the participants saw the studied images in the same temporal order as during encoding (middle panel), whereas the other half saw them in a fixed randomized order that differed from encoding (right panel). (Color figure online)

To eliminate the influence of intervening new items, we used a two-alternative forced-choice (2AFC) paradigm in which participants saw old images presented in the same order as during encoding, without any intervening new items as in the old/new recognition paradigms used in Experiments 1 and 2. We collected data from 50 participants per condition (100 participants in total), all residing in the United States and between 18 and 35 years old, using the Prolific online platform. Participants were compensated at a rate of $12 per hour. After providing informed consent for procedures approved by the Vanderbilt Institutional Review Board, each participant completed a study phase followed by a 2AFC recognition-memory test phase (see Fig. 2).

In the study phase, identical to Experiments 1 and 2, participants viewed 100 photographs of real-world objects, one exemplar from each of 100 distinct semantic categories. Each image subtended approximately 4.6° × 4.6° of visual angle, assuming an 80-cm viewing distance, and was centered on the screen during both study and test phases. Each trial began with a 1,000-ms fixation cross, followed by a picture displayed for 250 ms, after which a blank interval preceded the next picture. This continued until all 100 images had been shown.

In the test phase, each participant completed 100 trials, each presenting a pair of images consisting of one old and one new item. On each trial, one image appeared on the left and the other on the right, and participants indicated which image was old. The location of the old item was counterbalanced: It appeared on the left in half of the trials and on the right in the remaining half.

We divided our 100 participants into two groups consisting of 50 participants each. Both groups used the same 100 images (100 categories × 1 exemplar each) as their encoding set. They also shared the same 100 new images as the lures in their test phase. To manipulate the order of encoding, we made sure that the target images in test phase followed the same order as in the encoding phase, whereas for the other group, we made sure that the target images in the test phase were in a fixed, randomized order, different from the order during encoding.

The participants used the numbers on a keyboard to indicate their confidence and whether they thought the test stimulus was old or new. The number keys 1 and 2 indicated that the left item was old, with high and low confidence levels, respectively. The number keys 9 and 8 indicated that the right item was old, with high and low confidence levels, respectively. The stimuli were drawn from a published set of real-world objects (Brady et al., 2008), and programmed using the *jsPsych* package (de Leeuw, 2015).

## Results

**Fig. 3:**
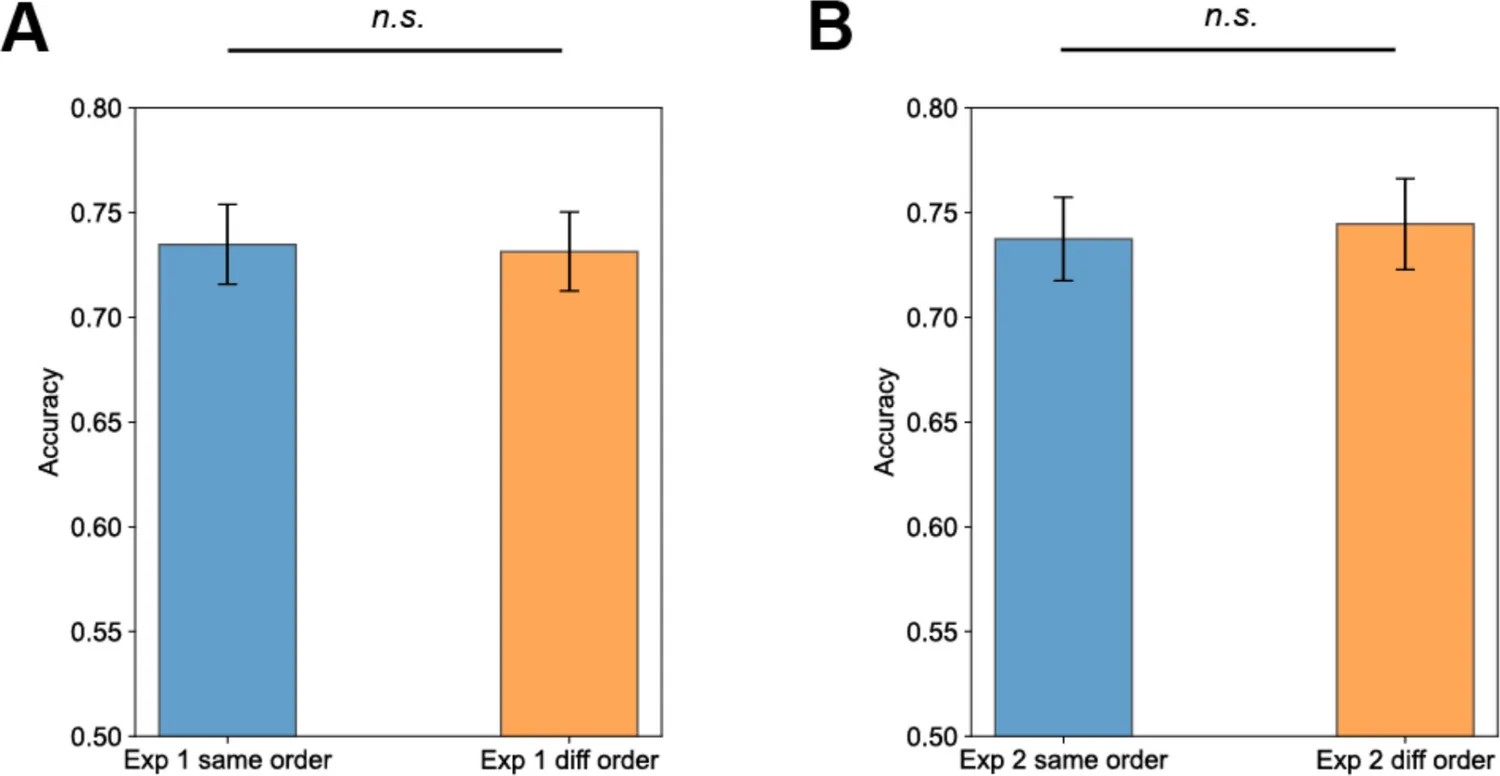
Recognition memory accuracy in Experiments 1 and 2. **A** Results from Experiment 1 showing mean accuracy from the test condition with the same order (blue) compared with the randomized order (orange). **B** Results from Experiment 2 with the same conventions as in A. (Color figure online)

We first examined whether following the same order as encoding would benefit overall memory-test accuracy. Contrary to the predictions of the *temporal order benefit hypothesis*, we found that participants had essentially identical accuracy during recognition-memory testing regardless of whether the test items followed the same order or a different order than participants had experienced during the study events, Experiment 1: *t*(98) = 0.15, *p* = 0.88, BF_10_ = 0.13; Experiment 2: *t*(98) = −0.28, *p* = 0.78, BF_10_ = 0.28 (see Fig. 3). Importantly, we had the sensitivity to detect differences if they had existed, as accuracies were below ceiling (Experiment 1 same: 73.48%; Experiment 1 different: 73.14%; Experiment 2 same: 73.73%; Experiment 2 different: 74.45%). Focusing on the hit rates for old items, we observed similarly below-ceiling performance for both same-order and different-order lists (Experiment 1 same: 64.66%; Experiment 1 different: 68.03%; Experiment 2 same: 68.16%; Experiment 2 different: 70.15%), showing that our lack of difference between conditions was not due to a ceiling effect. Moreover, we found no differences in reaction time for either old or new items, Experiment 1 old: *t*(98) = 0.49, *p* = 0.63, BF_10_ = 0.24; Experiment 1 new: *t*(98) = 1.30, *p* = 0.20, BF_10_ = 0.13; Experiment 2 old: *t*(98) = 0.97, *p* = 0.34, BF_10_ = 0.32; Experiment 2 new: *t*(98) = 1.20, *p* = 0.23, BF10 = 0.40. Therefore, our findings so far appear to disconfirm the predictions of the *temporal order benefit hypothesis* in that following the same study–test order did not improve overall performance.

**Fig. 4:**
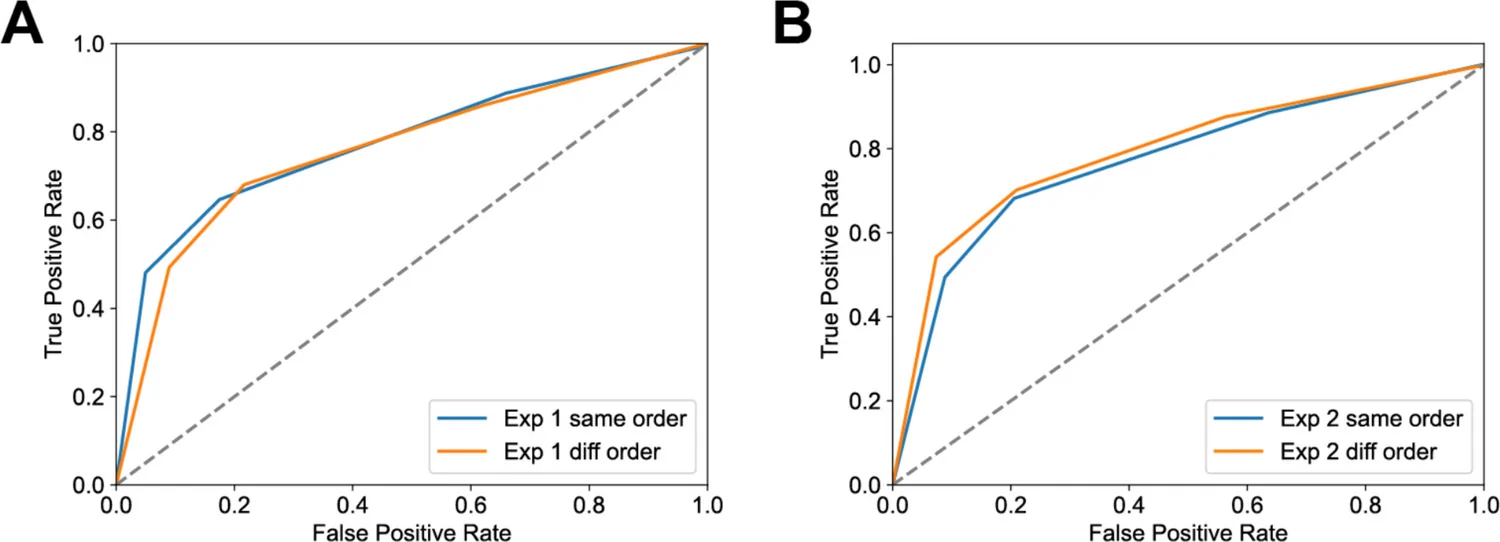
ROC curves in Experiments 1 and 2. **A** The ROC curves from Experiment 1 showing the same order of test in blue and the randomized test order in orange. **B** The ROC curves from Experiment 2 showing the same order of test in blue and the randomized test order in orange. (Color figure online)

Next, we wanted to make sure the true benefit was not obscured by a change in criterion between the two groups of participants. To address this possibility, we calculated *d′* measures of sensitivity, Experiment 1: *t*(98) = 0.22, *p* = 0.83, BF10 = 0.20; Experiment 2: *t*(98) = −0.52, *p* = 0.60, BF_10_ = 0.65, and beta measures of criterion, Experiment 1: *t*(98) = 0.67, *p* = 0.51, BF_10_ = 0.95; Experiment 2: *t*(98) = 0.15, *p* = 0.88, BF_10_ = 0.13. To provide another way to examine participants’ performance, we used participants’ confidence measures to computed their ROC curves. This allowed us to determine whether participants may have had benefits along some portions of the confidence scale, but not others (e.g., such as more high-confidence hits that are traded off by fewer low-confidence hits). Figure 4 shows the largely overlapping ROC curves from the same order and different order conditions (*p* values > 0.20).

**Fig. 5:**
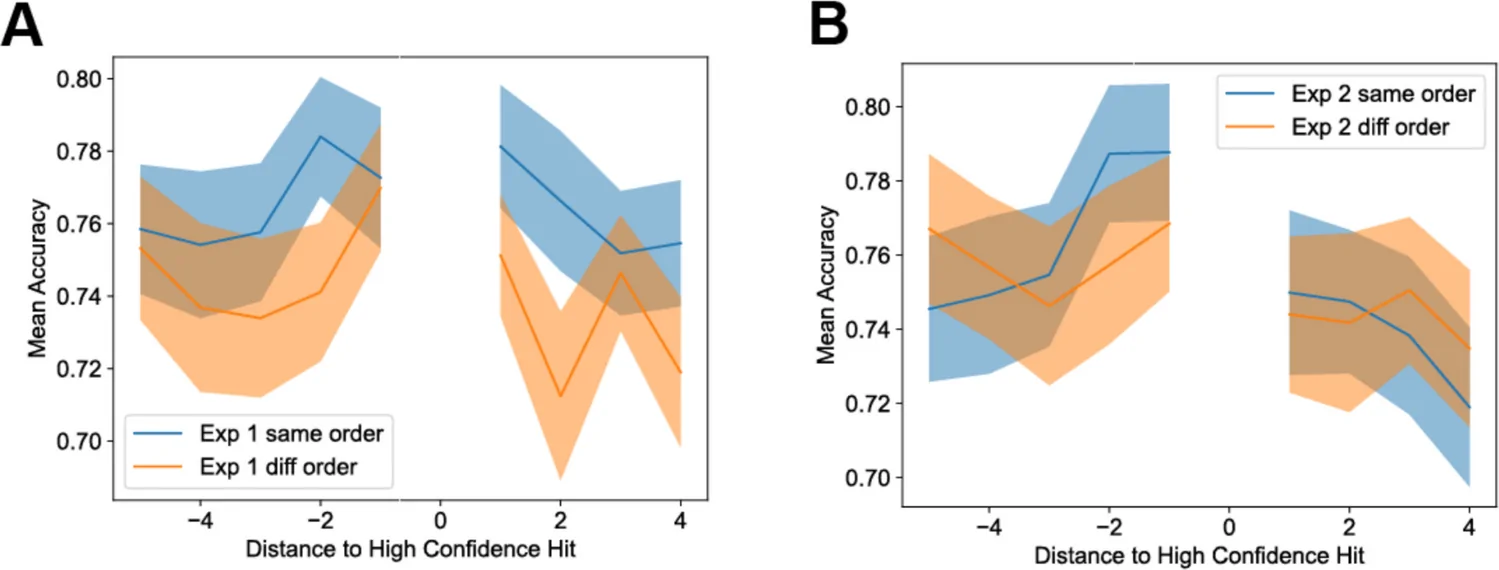
Item accuracies around high-confidence hits in Experiments 1 (left) and 2 (right). **A** Results from Experiment 1 showing mean accuracy conditional to high-confidence hit responses. **B** Results from Experiment 2 showing mean accuracy conditional to high-confidence hit responses. (Color figure online)

Our next set of analyses focused on the conditional-response probability plots used by Kahana and colleagues to study temporal organization in episodic memory across a range of different task and testing procedures (Healey et al., 2019). Figure 5 shows these plots. First, we note that accuracies around the high-confidence hit generally exhibited temporal grouping (i.e., peak accuracy flanking the 0 position) in both forward and backward directions, Experiment 1, forward: *t*(99) = 1.98, *p* = 0.04997; backward: *t*(99) = 2.03, *p* = 0.045; Experiment 2, forward: *t*(99) = 1.43, *p* = 0.16; backward: *t*(99) = 3.12, *p* = 0.002. However, the statistical comparisons suggest that the effect of keeping the temporal context the same at test did not significantly sharpen these peaks in either experiment (*p* values > 0.08). As Fig. 5 shows, the effect of order that approached significance was evident in Experiment 1, but we observed no such trend in Experiment 2.

**Fig. 6:**
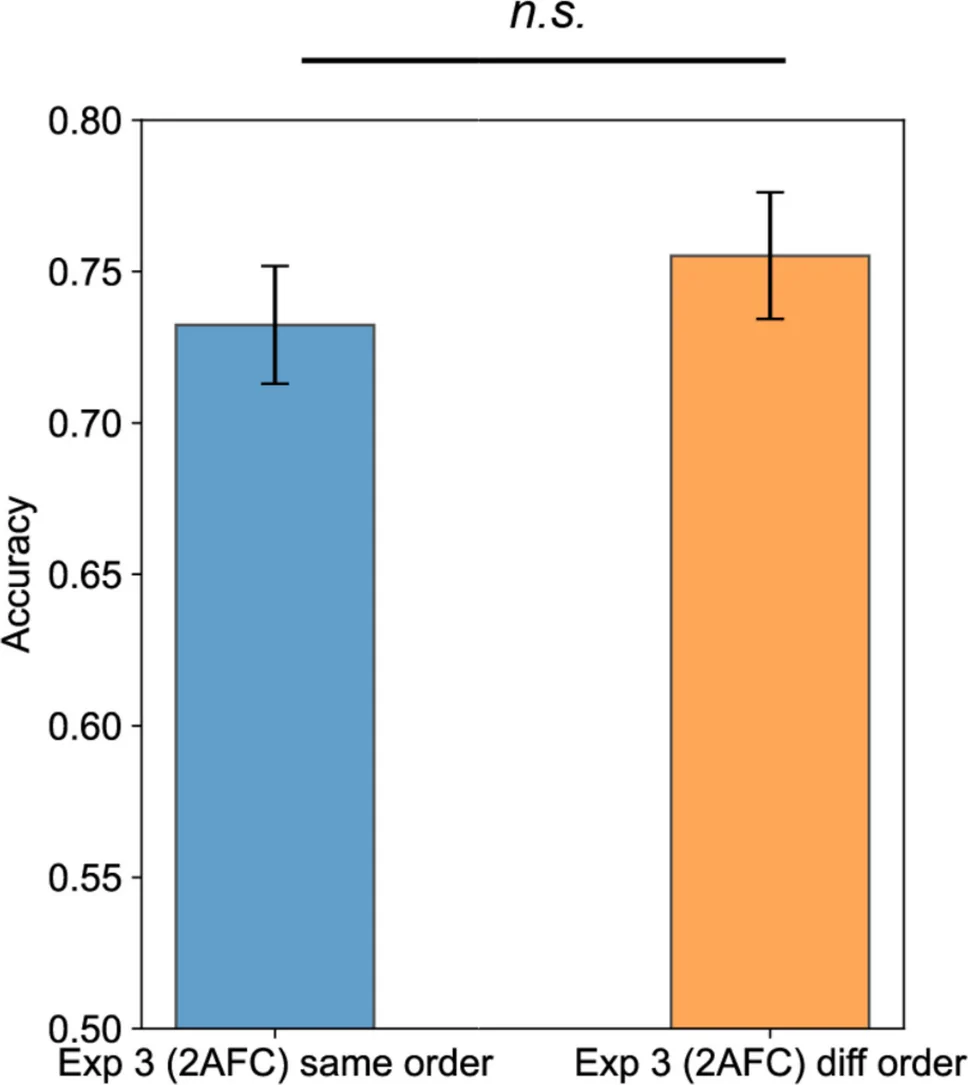
Recognition memory accuracy in Experiment 3 (2AFC). Results from Experiment 3 showing mean accuracy from the test condition with the same order (blue) compared with the randomized order (orange). (Color figure online)

Finally, to test whether any advantage of maintaining the same temporal order was diminished by intervening new items, we implemented a 2AFC version of the task in Experiment 3. In this design, each old item was presented directly alongside a new item, ensuring that the exact temporal order of the old items was preserved. Replicating the results of Experiments 1 and 2, we found that recognition accuracy was essentially identical regardless of whether the test sequence followed the same or a different order from the study phase, Experiment 3: *t*(92) = −0.92, *p* =.36, BF1_0_ = 0.32 (Fig. 6). Importantly, the task was sensitive enough to detect potential differences, as performance was well below ceiling (same-order: 73.23%; different-order: 75.52%). Moreover, we did not find differences in reaction time between same and different temporal order groups, Experiment 3: *t*(98) = −1.06, *p* = 0.29, BF_10_ = 0.36. Taken together, across three experiments, our findings consistently disconfirm the *temporal order benefit hypothesis*: preserving the study–test order did not improve recognition performance.

## Discussion

Our present experiments found that following the same temporal order did not benefit overall memory performance in a visual long-term memory-recognition task. Our findings challenge a prominent hypothesis regarding the nature of long-term memory storage. Namely, our findings seem contrary to predictions of the *temporal order benefit hypothesis*, which might be predicted from the general idea that matches between encoding and retrieval contexts improve memory retrieval (Kahana, 1996; Manning et al., 2011; Smith et al., 2022). This view predicts that overall accuracy should be higher when the test list follows the same order as the encoding phase than when it does not. Instead, we found similar performance when the temporal effects were aggregated across the entire session or examined with the fine-grained temporal sensitivity of conditional-response probabilities.

The absence of a temporal order benefit suggests that, although participants may form a temporal context for each item, item–item associations alone may not be sufficient to produce a retrieval advantage for a simple item-recognition task. One possibility is that temporal order supports retrieval primarily when lists are relatively short. In such cases, items remain active in working memory at test, allowing item–item associations to strengthen the activation of temporally adjacent items (Polyn & Woodman, 2025). Alternatively, temporal order may exert a stronger influence when it is directly relevant to the task. In our old/new recognition paradigm, temporal relationships between items do not provide a direct advantage for performance. In contrast, in tasks that require memory for event sequences, temporal order plays a more central role in organizing and reasoning about events. Consistent with this idea, prior work has shown that event boundaries can enhance temporal order memory when the resulting bindings help participants construct item-to-context associations (DuBrow & Davachi, 2013). A notable distinction between our study and previous investigations into the temporal contiguity effect in visual recognition memory is the utilization of a significantly longer list of items. Perhaps our participants encoded so many items that the utility of temporal structure diminished, and as a result they instead relied upon semantic grouping to structure their storage of the object representations (Morton & Polyn, 2017). This is a prediction that could be tested in future experiments by parametrically manipulating both temporal and semantic organization. It would be surprising to find large difference between storing approximately 65 images (Schwartz et al., 2005), and storing 100 images in the present experiments, given the evidence that visual long-term memory is so robust and can maintain a stable serial position curve across hundreds of intervening objects (Hollingworth, 2004). Furthermore, when participants encoded objects alongside irrelevant backgrounds, the background contexts significantly impaired object memory, indicating that context does not always facilitate long-term memory encoding (Evans & Wolfe, 2022). Clearly, future research is required to understand how the contextual structure of the environment is used to help store and differentiate our visual long-term memories. 

## Data Availability

Materials and data will be available through Open Science Framework (https://osf.io/ynvcm/).

## Code availability

Custom scripts used for stimulus presentation and analysis are available through Open Science Framework.

## Declarations

### Open practices statement

Materials and data are available through Open Science Framework (https://osf.io/ynvcm/).
